# Comparison of DMSA Scan 99 m and EC Scan 99 m in Diagnosis of Cortical Defect and Differential Renal Function

**DOI:** 10.5539/gjhs.v6n7p38

**Published:** 2014-09-18

**Authors:** M. Mohammadi-Fallah, M. Alizadeh, T. Ahmadi Lavin

**Affiliations:** 1 Nephrology and Kidney Transplant Research Center, Urmia University of Medical Sciences, Urmia, Iran; 2 Urmia University of Medical Sciences, Urmia, Iran

**Keywords:** cortical lesions, the differential renal function, EC, DMSA scan

## Abstract

**Introduction:**

Diagnosis of renal cortical lesions by radioisotopes in nuclear medicine is one of the most common techniques and procedures can be performed by different radiotracer. However, all these materials are accurate in determining kidney function, but there are differences between them in the field. The purpose of this study was to evaluate the effectiveness of EC scans compared with DMSA scan in the detection of cortical lesions and DRF.

**Methods:**

65 cases, which have been referred for various reasons, for DMSA scans were enrolled. Patients 1 week after DMSA scan with the previous consent of the EC being scanned. The results were compared in terms of convergence as well as sensitivity, specificity, positive and negative predictive value of EC with respect to the results of DMSA scan.

**Results:**

PPV of EC was 100%, negative predictive value of EC was 68.75%, sensitivity of EC was 90.74% and specificity of EC was 100% in the detection of cortical lesions. DMSA scan and EC convergence rates result in cortical lesions in our study was high.

**Discussion:**

We suggest EC scan as an alternative to reduce the cost of therapy and radiation, but considering the benefits of DMSA scan, it could remain the gold standard method of diagnosis.

## 1. Introduction

Diagnosis of renal cortical lesions by radioisotopes is one of the most common techniques in nuclear medicine. (Buyukdereli & Guney, 2006; Raja et al., 2012) This method can be performed by different radiotracer, such as technetium-99m dimercaptosuccinic acid (TC99m DMSA), technetium-99m diethylenetriamine pentaacetic acid (TC99m DTPA), technetium-99m mercaptoacetyltriglycine (TC99m MAG3), iodine 131 orthoiodohippurate (OIH) and more recently by technetium-99m ethylenedicysteine (TC99m EC). However, all these materials are accurate in kidney function, but there are differences between them in the field. The differences caused by biological features of radiotracer such as renal excretion mechanism, accumulation in renal cells, binding to plasma proteins and their plasma clearance (Raja et al., 2012). Currently renal scan by (TC99m DMSA) due to high levels of cortical accumulation is the most reliable and most sensitive test for detecting the location and extent of renal scars and renal parenchymal changes in comparison with sonography and intra venous pyelography (IVP). The method enables the calculation of the differential renal function (DRF) that shows functional changes in the damaged kidney (Kibar et al., 2003; Faravani et al., 2011). This material is mainly concentrated in cortical tubular cells of the kidney during function. Almost 90% of TC99m DMSA is bound to plasma proteins; it is limited in its glomerular filtration. The major problem with the use of this material is its higher radiation than any other materials that is mainly due to its high accumulation in renal tubules (Buyukdereli & Guney, 2006). Various labeled materials are used to evaluate the renal parenchyma. Because these materials are usually injected under the gamma camera for imaging and imaging is done during the first few minutes and before aggregation in the pelvis. Due to the small time interval for imaging, the image quality obtained is good, but the images are of low spatial resolution. However, renal radiotracers with higher leakage rate as TC99m EC creates better cortical images (Kibar et al., 2003). EC TC99m is a metabolite of ethylene cystine dimer. ECD is a new labeled marker with technetium. Due to faster clearance of TC99m EC absorbed radiation dose in patients is less and cortical image quality is better (Buyukdereli & Guney, 2006; Kibar et al., 2003). However, the accuracy of both methods in the evaluation of the renal cortex is high, but they differ in various surveys (Domingues et al., 2006). Given the lower radiation in using TC99m EC compared to DMSA scan and time-consuming compared with EC scan and diagnosis of obstructive lesions in this type of scan, seems to be the method of choice for imaging in the future. However, the sensitivity of this method in the study of cortical defects compared to DMSA scan in papers is still controversy. Papers have presented different results in this case (Buyukdereli & Guney, 2006; Kibar et al., 2003). The purpose of this study was to evaluate the effectiveness of EC scan compared with DMSA scan in the detection of cortical lesions and DRF. It is clear, if this hypothesis is confirmed, it is unnecessary to perform DMSA following EC scan to determine the DRF and cortical lesions and have many advantages for patients in terms of economy and health care.

## 2. Methods

Patients, who were referred to DMSA scan for different reasons, after informed consent, 65 patients were enrolled in this study and available information such as age, sex, presence of stones, hydronephrosis, stenosis, PUV, reflux and pyelonephritis were recorded in a check list. Patients underwent injecting labeled DMSA by nuclear medicine specialist and after one to two hours gamma camera imaging was performed. Patients, one week after DMSA scan with previous scan, underwent EC scan and immediately after injecting labeled EC material gamma camera imaging was performed and finally from evaluating DRF images and cortical lesions were calculated and analyzed. The results were compared in terms of convergence as well as sensitivity, specificity and positive and negative predictive value, EC scan with regarding the results of DMSA was obtained.

***Excluding criteria:*** Anatomic abnormalities such as duplex and ectopic kidney of patients with ARF and CRF, patients’ information from results of nuclear medicine section and during interview was obtained and recorded into the check lists. Finally, the results were analyzed using Spss 17 software (Majazi-Dalfard, 2013).

## 3. Results

In this study 65 candidate patients for renal function and cortical lesions referred to Imam Khomeini Hospital, Urmia were investigated, in terms of gender, 41 (63.1%) males with a mean age of 21.55±23.44 and 24 (36.9%) females with a mean age of 18.69±27.92 years. Patients’ age was 1 to 68 years. In this study, based on DMSA scans of 65 patients, 54 patients (83.1%) had renal scarring, and 11 patients had no scar that EC scan of the 54 patients in the 49 cases of scar was detected and in 5 cases (9.27%) was not detected. Of 54 cases that were detected scar, detecting reason was as follows: 2 patients with a history of numerous kidney stone lithotripsy (3.7%), 1 patient with neurogenic bladder (1.8%), 7 patients with reflux (12.96%), 4 cases with pyelonephritis + stone (7.4%), 11 cases with UPJO (20.3%), 8 cases with antler stone, (14.8%), 2 cases with pyelonephritis (3.7%), 3 cases with agenesis (5.55%),3 cases with ureteral stone (5.55%) and3 cases with narrowing of the distal ureter (5.55%), 3 cases with pyelonephritis + VUR (5.55%) and 2 cases with agenesis + VUR (3.7%) and 5 cases with hydronephrosis (9.25%) (Table 1).

**Table 1:** Absolute and relative frequency of diagnosed scar cases in the study population

Scar diagnosis reason	Frequency	Percentage
Kidney stones (lithotripsy experience)	2	3.7
Neurogenic bladder	1	1.8
Reflux 7	7	12.96
Stone + pyelonephritis	4	7.4
UPJO	11	20.3
Stone antler	8	14.8
Pyelonephritis	2	3.7
Agenesis	3	5.55
Ureteral stone	3	5.55
Distal ureteral stricture	3	5.55
Pyelonephritis + VUR	3	5.55
+ VUR Agenesis	2	3.7
Hydronephrosis	5	9.25
Total	54	100

Surveying scar by DMSA Scan showed that, of the 54 cases of scar 100% has been detected and of 11 cases that did not have scar, the scar was negative (Table 3).

**Table 2:** Distribution of scar detected by EC Scan

EC Scan	Have	Do not have	
scar	49(90.7%)	0(0%)	49
Non-scar	5(31.3%)	11(68.8%)	16
Total	54(83.1%)	11(16.9%)	65

**Table 3:** Distribution of scar detected by DMSA Scan

DMSA SCAN	Scar	

	Have	Do not have
scar	54(100%)	0(0%)
Non-scar	0(0%)	11(100%)
Total	54(83.1%)	11(16.9%)

Given that in our study gold standard is considered by DMSA and investigated as the objectives of DMSA scan and EC scan together. Compared results showed that, of 45 detected scars 49 cases (90.7%) has been positive and 5 cases has not been detected. Of 11 cases that had not scar 100% has been detected negative. According to the Fisher Exact Test there is a significant difference between DMSA scan and EC scan methods and had 100% specialty, 90.74% sensitivity, 100% positive predictive value and 68.75% negative predictive value (Table 4).

**Table 4:** Comparing DMSA and EC scan in detecting scar

DMSA SCAN	EC Scan	

	Have	Do not have
Scar	49(90.7%)	5(9.3%)
Non-scar	0(0%)	11(100%)
Total	49(75.4%)	16(24.6%)

DRF of 60 patients, according to the results of DMSA Scan in the right kidney was equal to 16.11±52.25 and DRF of left kidney was 16.38±48.05. DRF of 60 patients, according to the results of EC Scan in the right kidney was equal to 16.04±53.19 and DRF of left kidney was 16.04±46.80 (Table 5).

**Table 5:** Mean and standard deviation of the DRF right kidney and left kidney in two ways of DMSA Scan and EC Scan

	The mean and standard deviation of DRF	

	Right	Left
DMSA Scan	16.11±52.25	16.38±48.05
EC Scan	16.04±53.19	16.04±46.80

There is a high correlation coefficient and Pearson correlation between the two methods between DRF of right kidney (r=0.93 and P = 0.001). There is a high correlation between the two methods between DRF of left kidney by two DMSA scan and EC scan. (r=0.95 and p=0.001). In response to Goal 6: Convergence rate results of EC scan and DMSA scan according with kappa test with 0.76% of convergence exist between the two methods in terms of DRF and scar.

## 4. Discussion

Diagnosis of renal cortical lesions by radioisotopes in nuclear medicine is one of the most common techniques and procedures can be performed by different radiotracer. However, all these materials are accurate in determining kidney function, but there are differences between them in the field. Currently renal scan by (TC99m DMSA) due to high levels of cortical accumulation is the most reliable and most sensitive test for detecting the location and extent of renal scars and renal parenchymal changes in comparison with sonography and intra venous pyelography (IVP). The most important problem in using this material is its higher radiation to kidney compared with other materials that is mainly because of concentration in renal tubular. Renal radiotracers with higher leakage rate as TC99m EC creates better cortical images. Due to faster clearance of TC99m EC absorbed radiation dose in patients is less and cortical image quality is better. However, the accuracy of both methods in the evaluation of the renal cortex is high, but they differ in various surveys. Given the lower radiation in using TC99m EC compared to DMSA scan and time-consuming compared with EC scan and diagnosis of obstructive lesions in this type of scan, seems to be the method of choice for imaging in the future. However, the sensitivity of this method in the study of cortical defects compared to DMSA scan in papers is still controversy. Papers have presented different results in this case. The purpose of this study was to evaluate the effectiveness of EC scan compared with DMSA scan in the detection of cortical lesions and DRF. Positive predictive value of EC was 100%, negative predictive value of EC was 68.75%, and sensitivity was 90.74%, specificity of EC was100% in the detection of cortical lesions. According to the results of our study and that the convergence of the DMSA and EC scans was high in detecting cortical lesions, it can be suggested that Tc-99m EC scans that have less radiation is used alone to assess cortical lesions and to determine the DRF. Given that Tc-99m EC images show the DRF rate with high contrast and accuracy in patients, it can make possible to detect most problems of the renal parenchyma. We can use this method as an alternative method for reducing the dose of radiation and the cost of radiation treatment but due to the well-known benefits, DMSA scan remains the gold standard method of diagnosis.

**Comparing previous studies with this study:** A similar study conducted in 2006 by Buyukdereli et al. (Buyukdereli et al., 2006) in the results of renal scintigraphy Tc-99m EC and Tc-99m DMSA, 99 and 97 focal lesion was found. High positive correlation between the diagnoses was obtained in the DRF. (R = 0.91, P = 0.001). Average of DRF in the left kidney in Tc-99m EC and Tc-99m DMSA images was 19.1 ± 45.8 and 20.4 ± 45.0, respectively. In our study, the correlation between the two diagnostic methods in DRF on the right kidney was obtained (r=0.93 and P = 0.001). Left kidney DRF was (r=0.95 and P=0.001).

As a study conducted by Domingues et al. in 2006, also in our study the renal function was measured by Tc-99m EC, values obtained by Tc-99m DMSA showed no significant difference (Domingues et al., 2006). As a study conducted by Raja et al. in 2012 (Raja et al., 2012) Tc-99m DMSA was able to detect cortical scars in 41 kidneys, while Tc-99m EC was able to detect 41 cases out of 29. Renal activity of both methods was well correlated. (R was 0.95 for the left kidney and for the right kidney was 0.94). Conclusions noted that the sensitivity of renal scintigraphy with Tc-99m EC compared to Tc-99m DMSA in renal cortical review of scar was lower (70% compared to 100%). In our study the renal function of both methods showed good correlation and sensitivity and specificity obtained in our study of Tc-99m EC was 90.74% and 100%, respectively. In our survey the sensitivity for the detection scar was higher. As a study conducted by Kibar et al. in 2003 (Kibar et al., 2003) Tc-99m DMSA had detected more renal parenchymal lesions and the sensitivity and specificity obtained in our study for Tc-99m EC was 90.74% and 100% respectively. In Kibar et al study there was a very close convergence in DRF of the two methods (r=0.99). The Tc-99m DMSA was detected more renal parenchymal lesions and the sensitivity and specificity of Tc-99m EC was 92.6% and 100%, respectively that was very similar to our study. In a study conducted by Dr. Elham Faravani et al. in 2008 (Faravani et al., 2011) in our study also there was a close convergence between the two methods of DRF results. A similar study conducted by Atasever et al. in 2004 (Atasever et al., 2004) and EC scan with a sensitivity of 82.5% successfully detect moderate and severe cortical lesions, but was less successful in detecting mild lesions (sensitivity 60%) in the conclusion noted that EC scan detect most of the cortical lesions. The sensitivity of this method is dependent on the severity of the injury. The EC provided reliable information about the measurement of DRF. In our study also EC scan had detected most of the cortical lesions, but investigates whether the sensitivity of this method is dependent on the severity of the lesion was not performed. The EC provided reliable information about the measurement of DRF.

## 5. Suggestions

It is recommended that in the future studies larger numbers of cases use to achieve results that are more consistent with a normal distribution. In future studies, other methods of scanning in order to achieve the best and most beneficial methods are compared to each other. In future studies, severity of the scar on determining the ability to identify the scan should be considered.
